# Long-term clinical outcomes in a cohort of patients with solitary plasmacytoma treated in the modern era

**DOI:** 10.1371/journal.pone.0219857

**Published:** 2019-07-23

**Authors:** F. A. Sharpley, P Neffa, F. Panitsas, J. Kothari, M. Subesinghe, D. Cutter, R. Shcolnik Szor, G. Aparedcida Martinez, V. Rocha, K. Ramasamy

**Affiliations:** 1 Oxford University Hospital NHS Foundation Trust, Oxford, United Kingdom; 2 Division of Hematology, Hospital das Clinicas of the University of São Paulo, São Paulo, Brazil; 3 NIHR BRC Blood Theme, Oxford, United Kingdom; 4 Department of Cancer imaging, School of Biomedical Engineering and Imaging Sciences, King’s College, London, United Kingdom; George Washington University, UNITED STATES

## Abstract

**Background:**

The risk of recurrence of solitary plasmacytoma (SP)/progression to MM is well established, but patient, imaging and treatment factors influencing risk of progression require further evaluation.

**Methods:**

This is a retrospective analysis of 66 SP patients (23 UK, 43 Brazil) diagnosed 1989–2016. Patient baseline characteristics were recorded. The incidence of progression to MM was calculated, including biochemical and imaging findings and the treatment modality received. Survival estimates were determined by Kaplan-Meier analyses.

**Results:**

With a median follow-up of 53.6 months the 5 year overall survival (OS) was 90.7% (95%CI 79–96%). The median progression free survival (PFS) from diagnosis was 61 months. Cumulative incidence of progression to MM was 49.9% at 5 years (95% CI 35.6–62.6%) and was significantly higher with bone plasmacytoma (47.2%, 95%CI 31.9–61.1%), than an extramedullary location (8.3%, 95%CI 0.4–32.3%, Gray test p = 0.0095)). The majority of patients with solitary bony plasmacytoma (SBP) received radiotherapy (RT) (51/53, 96.2%) whereas most extramedullary cases were treated with surgical resection (7/13, 53.8%). A small proportion of SBP patients received additional upfront chemotherapy, with 5/6 in remission after a median follow-up (FU) of 10 years. The diagnostic yield of surveillance functional FU imaging without other indications of relapse/progression was low. The positive predictive value of functional FU imaging was high but with a low negative predictive value, especially in cases of suspected relapse/progression.

**Conclusion:**

Our data suggests functional imaging should be used if clinical suspicion of relapse/progression, rather than a routine surveillance tool, and upfront adjuvant chemotherapy is worthy of prospective evaluation.

## Introduction

Solitary plasmacytomas (SP) are rare, comprising less than 5% of all plasma cell neoplasms. The International Working Myeloma Group (IMWG)[1] has defined three discrete categories of SP: solitary plasmacytomas (SP), extramedullary plasmacytomas (EMP) and solitary plasmacytoma with bone marrow (BM) involvement. Patients may present with bony pain, neurological symptoms and/or pathological fracture in the case of a SP or epistaxis, rhinorrhoea and nasal obstruction in the case of an upper respiratory tract EMP, which is the most commonly affected site.

### Diagnosis of solitary plasmacytomas

The diagnosis of a SP relies upon histological evidence of a tumour consisting of monoclonal plasma cells, with a plasma cell percentage in the BM of <10%, along with imaging confirmation of a solitary tumour with no other evidence of organ or tissue damage. It is known that conventional staging with skeletal X-ray survey will understage some patients.[2] As a result, some patients will be miscategorised as SP and may receive radical radiotherapy (RT), the treatment of choice for SP, rather than benefitting from systemic chemotherapy to treat symptomatic myeloma. To improve the accuracy of diagnosis of SP, there has been a shift towards whole body imaging techniques.

Magnetic resonance imaging (MRI) remains the gold standard imaging modality for detecting BM infiltration at an early stage, prior to bone destruction, particularly in the axial skeleton. Newer MRI protocols enable whole body imaging techniques (WB-MRI) to improve coverage of the appendicular skeleton, whilst diffusion weighted sequences (DW-MRI) improve specificity.[3] 2-deoxy-2-[^18^F]fluoro-D-glucose (FDG) positron emission tomography-computed tomography (PET-CT) has emerged as a powerful hybrid imaging tool both for diagnosis of plasmacytomas, and also for monitoring treatment response, with a growing body of data suggesting the benefit over conventional MRI in the setting of myeloma (MM).[4] In Brazil access to FDG PET-CT is not widely available, and hence ^99m^Tc-Sestamibi (^99m^Tc-MIBI) scans are used for diagnosis and monitoring of some patients. Although not as accurate as FDG PET-CT for detecting focal lesions, ^99m^Tc-MIBI can be better than FDG PET-CT at detecting diffuse marrow involvement. [5] ^99m^Tc-MIBI is an inexpensive and rapid technique for whole-body evaluation enabling its wider availability in developing countries like Brazil.[5]

### Risk of progression to myeloma

Patients with SP can progress to overt MM at a rate of approximately 40% to 50% over 5 years, with lower rates of progression reported for EMP.[6] The Rare Cancer Network described the natural history of 258 patients with SPs and found the median time to MM progression was 21 months (2–135).[6, 7] One possible explanation for this wide variation in progression free survival (PFS) is that this study was reported in 2006, when the primary imaging modality was skeletal survey, and some patients with MM may have been wrongly classified as plasmacytoma resulting in a relatively short PFS. Despite this, the study still reflects the fact that SP patients are a heterogeneous group, with some progressing early to MM, whilst others remain disease free for many years or are effectively cured.

A number of risk factors are postulated as predictors of disease progression. Warsame et al. (2012) identified the presence of excess clonal plasma cells in BM greater than or equal to 5%, and delivered radiotherapy dose as prognostic factors for risk of progression.[8] Whilst Knobel et al. (2006) reported patient age as a risk factor for progression[9] and Tasang et al. (2001) reported tumour size to be a predictive factor.[10] Other recognized risk factors for progression to MM include abnormal serum free light chains (sFLC) ratio at diagnosis, and M-protein persistence post RT.[11] Both Spanish and UK groups have independently demonstrated that the presence of aberrant plasma cells on flow cytometry of the BM can identify patients with SPs at high risk for progression to active MM. ^[^^12^^–^^14^^]^

The use of imaging methods to assess patients with SP at risk of progression has also been explored.[15, 16] Fouquet et. al (2014) identified baseline FDG PET-CT features as a strong predictive factor for risk of progression, but some of these features would now be classified as MM.[17] Whether functional imaging findings post-therapy reliably predict risk of progression remains unknown, and whether SUVmax can be used as a biomarker of risk remains unknown.

We retrospectively analysed a large cohort of patients diagnosed more recently to understand the natural history of this condition and validate the known risk factors for progression to myeloma. All our patients had advanced imaging at diagnosis and so we examined if features of functional imaging would serve as biomarker for disease progression.

## Materials and methods

Sixty-six patients with SP were identified, (23 UK, 43 Brazil). All patients had a histological diagnosis of SP or EMP as defined by IMWG criteria.^1^ Patient baseline characteristics were recorded including the size and location of their plasmacytoma, imaging findings in addition to paraprotein/light chain and BM results.

All patients in the UK cohort had imaging by either conventional skeletal survey (6/23), conventional MRI (7/23), FDG PET-CT (17/23: before any intervention N = 6, after excision/incision biopsy N = 9, after RT N = 4), or by all three imaging techniques. The Brazilian cohort was imaged by skeletal survey (81.3% = 35/43), CT (60.4% = 26/43) MRI (55.8% = 24 /43) and MIBI (27.9% = 12/43). A single radionuclide radiologist retrospectively reviewed all images on patients who had FDG PET-CT and reported the number and size of plasmacytomas seen on each imaging modality to assess what proportion of patients were subsequently upgraded to MM and whether their treatment pathway had changed.

A proportion of patients had follow-up FDG PET-CT or ^99m^Tc-MIBI imaging. We assessed the proportion progressing to MM, the biochemical and BM results at the time of imaging and the treatment they received. Disease progression was defined as the detection of new plasmacytoma on follow-up FDG PET-CT imaging or by IMWG diagnostic criteria.

Statistical analysis was performed using Stata (StataCorp. 2017. Stata Statistical Software: Release 11.2. College Station, TX: StataCorp LLC). Progression free survival (PFS) was defined as time from date of diagnosis until evidence of disease progression (recurrence of SP progression to multiple plasmacytomas/MM) or death, and overall survival (OS) from time of diagnosis until death from any cause. Both were determined by survival estimates using Kaplan-Meier analyses. We used PFS measured from date of radiotherapy to examine effect of radiotherapy dose on PFS outcome, in order to avoid any selection bias introduced by treatment delays. Cumulative incidence of disease progression (multiple plasmacytomas/MM), and the Gray test for univariate incidence comparisons, were both performed using the easy-to-use software (EZR, or Easy R). Because of small patient numbers and small number of events as well as missing data, multivariate analysis was not attempted. In view of the retrospective and often incomplete data collection, all analyses were performed for descriptive purposes and no correction for multiple testing was made. This study was approved by the Institutional Review Board at Oxford University Hospitals NHS Foundation Trust (Clinical Governance Committee).

## Results

The median patient age was 60 years (range 19–82). There was no difference in age between the cohorts (60 years UK, 58 years Brazil, p = 0.81) (see Table 1). Gender, location (SBP/EMP) and BM involvement also did not differ between the two cohorts. Race distribution differed between the groups (44.2% of Brazilian cohort were African origin, 100% UK White-Caucasian.)

**Table 1:** A comparison of baseline patient characteristics with solitary plasmacytoma (SP) from Brazil and the UK.

	Brazil	UK	Comparison	Total
**Number of patients**	43	23		66
**Gender**	Male 28 (65.1%), Female 15 (34.9%)	Male 17 (73.9%), Female 6 (26.1%)	Pearson chi(2) p = 0.47	Male 45 (68.2%), Female 21 (31.8%)
**Age**	median 58 years, IQR 48–66, range 18–78	median 60 years, IQR 44–72, range 19–82	Mann-Whitney p = 0.81	Median 59.5 years, IQR 35–75, range 18–82
**Location**	Bone 35 (81.4%), extramedullary 8 (18.6%)	Bone 18 (78.3%), extramedullary 5 (21.7%)	Fisher's exact p = 0.76	Bone 53 (80.3%), extramedullary 13 (19.7%)
**Size**	65 mm, IQR 33–90 (N = 35)	65mm, IQR 32–80 (N = 11)	Mann-Whitney p = 0.73	65mm, IQR 33–90 (N = 46)
**Bone marrow plasma cell infiltration at diagnosis**	median 0%, IQR 0–2%, range 0–9.2%	median 4%, IQR 0–5%, range 0–8%	Mann-Whitney p = 0.065	median 0.1%, IQR 0–2.9%, range 0–9.2%
**Year of diagnosis**	median 2011, range 2000–2015	median 2012, range 1989–2016		median 2012, range 1989–2016
**Radiotherapy**	No 5 (11.6%), Radical 29 (67.4%) Palliative 9 (20.9%)	No 4 (17.4%), Radical 17 (73.9%), Palliative 2 (8.7%)	Fisher's exact p = 0.44	No 9 (13.6%), Radical 46 (69.7%), Palliative 11 (16.7%)
**Time from diagnosis to RT completion**	Median 148 days, IQR 96–232 (N = 38)	median 64 days, IQR 30–127 (N = 18)	Mann-Whitney p = 0.0006	median 123 days, IQR 64–202 (N = 56)
**Chemotherapy 1st line**	N = 5, 11.6%	N = 1, 4.35%	Fisher's exact p = 0.66	N = 6, 9.1%
**follow-up**	Median 57.4 months, IQR 39.4–80	Median 39.6 months, IQR 25.3–59	Mann-Whitney p = 0.11	median 53.6 months, IQR 34.4–72.2
**Overall Survival at 5 years**	92.3%, 95%CI 77.8–97.4%	86.4%, 95%CI 77.8–97.4%	log-rank p = 0.56	90.7%, 95%CI 79–96.1%
**PFS at 5 years**	54.5%, 95%CI 37.7–68.6%	37.4%, 95%CI 13.4–61.8%	log-rank p = 0.58	50.1%, 95%CI 36.1–62.7%
**Cumulative incidence of progression to MM at 5 years**	45.5%, 95%CI 29.1–60.4%	62.6%, 95%CI 28.9–83.8%	Gray p = 0.49	49.9%, 95%CI 35.6–62.6%

RT = radiotherapy; PFS = progression free survival; MM = multiple myeloma; IQR = inter-quartile range

The SP was a SBP in 83% of cases (53/66) and EMP in 19.7% of cases (13/66). The most common SP location was in the thoracic spine (25.76%). The least common location for a SP was the ribs (3.03%). (See Table 2). The median plasmacytoma size at diagnosis was 60mm (range 19-220mm).

**Table 2:** Location of solitary plasmacytoma (SP).

SP location	Patients (n/ %)
Skull	4 (6.1)
C-spine	6 (9.1)
T-spine	17 (25.8)
L-spine	6 (9.1)
Pelvis	12 (18.2)
Ribs	2 (3.0)
Long bones	6 (9.1)
EMP:	13 (19.8)
Liver	1
Retroperitoneal	1
LN	2
Thyroid	1
Skin	1
Intestinal	2
Eye	1
Upper Airway	3
Meninges	1
Total	66

C-spine = cervical spine, T-spine = thoracic spine, L spine = lumbar spine, EMP = extramedullary plasmacytoma.

Measurement of sFLC at diagnosis was available in 20 patients (all UK). sFLC ratio was abnormal in 10 cases (50%) with kappa chain excess in 8 cases (median 212 mg/L, range 23.2–989 mg/L) and lambda chain excess in 2 cases (89.9 mg/L and 1060 mg/L). Patients with abnormal sFLC ratio at diagnosis tended to have larger tumours (median size 65mm vs 18.5mm) although difference was not significant probably because of small numbers. Baseline beta2 microglobulin (b2m) levels were available for 40 patients. Median value was 1.87 mg/L, IQR 1.6–2.3 mg/L, range 0.9–7.4 mg/L. Based on ROC curve analysis, 1.9 mg/L was selected as threshold to split cases into two groups with high and low b2m. No statistical effect of binary b2m on outcomes could be detected, although SBP patients with higher baseline b2m levels appeared to have more events (data not shown). Data on BM examinations at diagnosis could be retrieved in 56/66 cases. Highest reported percentage of plasma cell infiltration on BM aspirate or trephine biopsy was used. All patients had ≤10% BM involvement at initial presentation. Median percentage was <1%, range 0–9.2%. Patients with >1%, compared with ≤1% plasma cells in the BM, showed a trend towards less favourable progression free survival (44.7 months vs 73.6 months respectively, P = 0.099) with a higher 4 year cumulative incidence of multiple myeloma (59.6% vs 43.8% respectively, P = 0.046)) and a poorer 4 year OS (65.9% vs 100% respectively, P = 0.045). Patients with EMP had significantly lower BM plasma cell involvement (Mann-Whitney p = 0.03).

**Fig 1:**
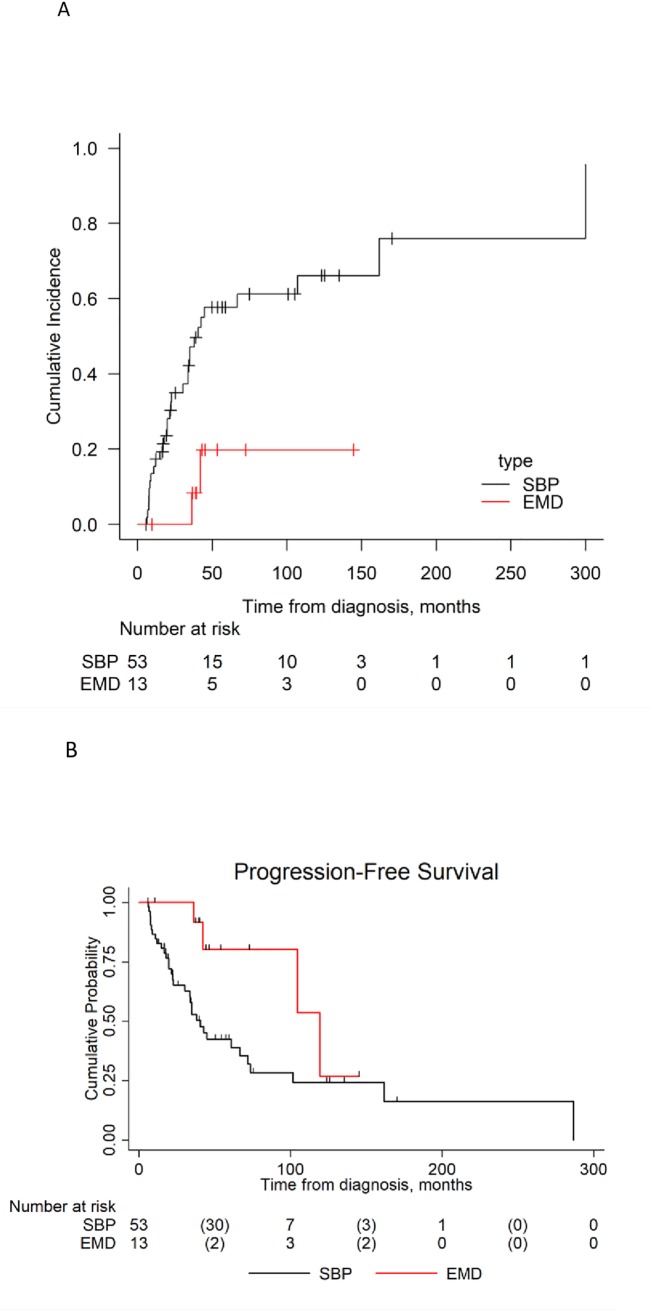
**(A)** Cumulative Incidence of Multiple Myeloma (MM) by Solitary plasmacytoma type: EMD = extramedullary disease/plasmacytoma **(B):** The effect of plasmacytoma type on progression free survival (PFS): SBP = solitary bony plasmacytoma, EMD = extramedullar plasmacytoma.

Median FU was 53.6 months (range 5.8–327 months), with 9 deaths during this time. Estimated OS at 5 years is 90.7% (95% CI 79–96.1%). There was no significant difference in OS between the two centres with Kaplan-Meier curves overlapping. Cumulative incidence of progression to MM at 3 years was 37.2% for the whole cohort (95% CI 24.8–49.6%), 47.2% (95% CI 31.9–61.1%) for SBPs versus 8.3% (95% CI 0.4–32.3%) for EMPs (Gray test p = 0.0095). Median PFS of both groups combined was 5 years 1 months (UK—42 months, Brazil– 67 months), with 31 patients 47 alive in first remission, 7 patients (11%) having recurrent plasmacytoma (all in a different location) and 28 patients (42.4%) progressing to MM in this time. Estimated PFS was 50.1% at 5 years (95% CI 36.1–62.7%). Within the SBP cohort, 21/53 (39.6%) remain in first remission, 6 relapsed with SP (11.3%) and 26 (49.1%) progressed to MM. This compares with 10/13 (77%) with initial EMP remaining in first remission, 1 had recurrent plasmacytoma (7.7%) and 2 developed MM (15.4%), (Fig 1A). Median PFS was 40.6 months for SBPs and 119.4 months for EMP. PFS was 52.8% (95% CI 37.3–66.1%) for SBPs and 91.7% (95% CI 53.9–98.8%) for EMPs at 3 years. Hazard ratio for PFS of EMP vs SBP was 0.35 (95% CI 0.12–0.99, p = 0.048).

By univariate analysis no effect of size of SP at diagnosis (>60mm vs ≤60mm) on progression to MM could be detected (Gray test p = 0.34), (see Fig 1B). Abnormal baseline sFLC ratio did not appear to predict PFS (log-rank p = 0.52) or MM incidence (Gray p = 0.51), but numbers are small.

### Treatment for plasmacytoma

RT was the treatment of choice for the vast majority of patients. In total 57/66 patients (86.4%) received RT. The median time from diagnosis to completion of RT was 123 days (IQR 64–202) and RT was delivered faster in SBP cases (median 112.5, IQR 59–188 days) than in EMP cases (median 229.5, IQR 150–238 days), Mann-Whitney p = 0.0235). In patients with SBP, delivery of RT was not delayed by surgical treatment (Mann-Whitney p = 0.34; surgically treated median 106 days, IQR 50–168; non-surgically treated median 119 days, IQR 76–189). The dose of RT was available in 47/ 57 patients. The RT dose range was 8-54Gy, 45 patients (68.2%) were given a “radical” dose (≥40Gy) and 12 patients (68.2%) a “palliative” dose (<40Gy, N = 12, 18.2%). The median “radical” RT dose was 45 Gy (40–54) and median “palliative” RT dose was 30 Gy (8–38). In the SBP patient cohort the majority 39/53 (73.6%) received radical RT, 12 (22.6%) palliative RT and 2 (3.8%) no RT. In the EMP cohort 6/13 (46.2%) received radical RT whilst 7 were treated with surgical excision only. Among patients who received radical RT, a higher dose (median 45Gy, range 40–54) was delivered to bone locations (N = 29) compared to soft tissue locations (N = 6 median 40Gy, range 40–45, Mann Witney p = 0.005). Patients who were treated with radical RT were younger (median age 57 years, range 18–82) than patients who received reduced dose RT (median age 62.5 years, range 35–78) or no RT (median 64 years, range 48–75), but this was not statistically significant (p = 0.27). There was no difference in the age distribution between SBP and EMP cases.

**Fig 2:**
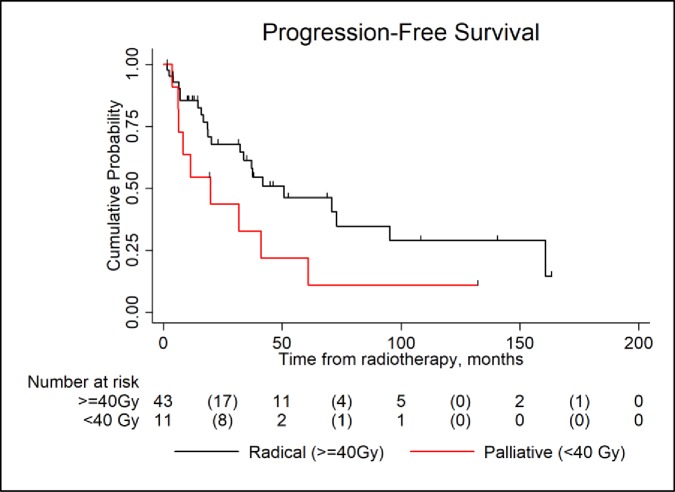
How radiotherapy dose (radical >40Gy, or palliative <40Gy) affects progression free survival (PFS).

Patients who received less than 40 Gy (palliative dose) appeared to have less favourable PFS calculated from time of RT (HR 2.11, 95% CI 0.97–4.6, p = 0.06)), (Fig 2). However, RT dose level seemed to lose its predictive value when analysis was adjusted for initial tumour size (>60mm) and location (SBP vs EMP). Unmeasured confounders (frailty, comorbidities etc) affecting therapy decisions may also account for this difference in PFS outcomes.

The second most commonly used treatment modality was surgical excision. A complete or partial excision of the tumour was performed in 37/66 (56.1%) of patients. This was with the intent of confirming a diagnosis and potentially excising a new tumour of unknown nature (mainly for EMP). Surgery was also undertaken pre-emptively in cases of potential pathological fractures of the long bones or to provide relief or stabilisation in cases of threatened or proven spinal cord compression (Table 3).

**Table 3:** The number of patients receiving surgical resection based on solitary plasmacytoma location.

Location	No resection	Surgical Resection
Skull	2	2
C-spine	2	4
T-spine	6	11
L-spine	5	1
Pelvis	9	3
Ribs	1	1
Long Bones	3	3
EMD	1	12
Total	29	37

C-spine = cervical spine, T-spine = thoracic spine; L-spine = lumbar spine, EMD = extramedullary disease.

**Fig 3:**
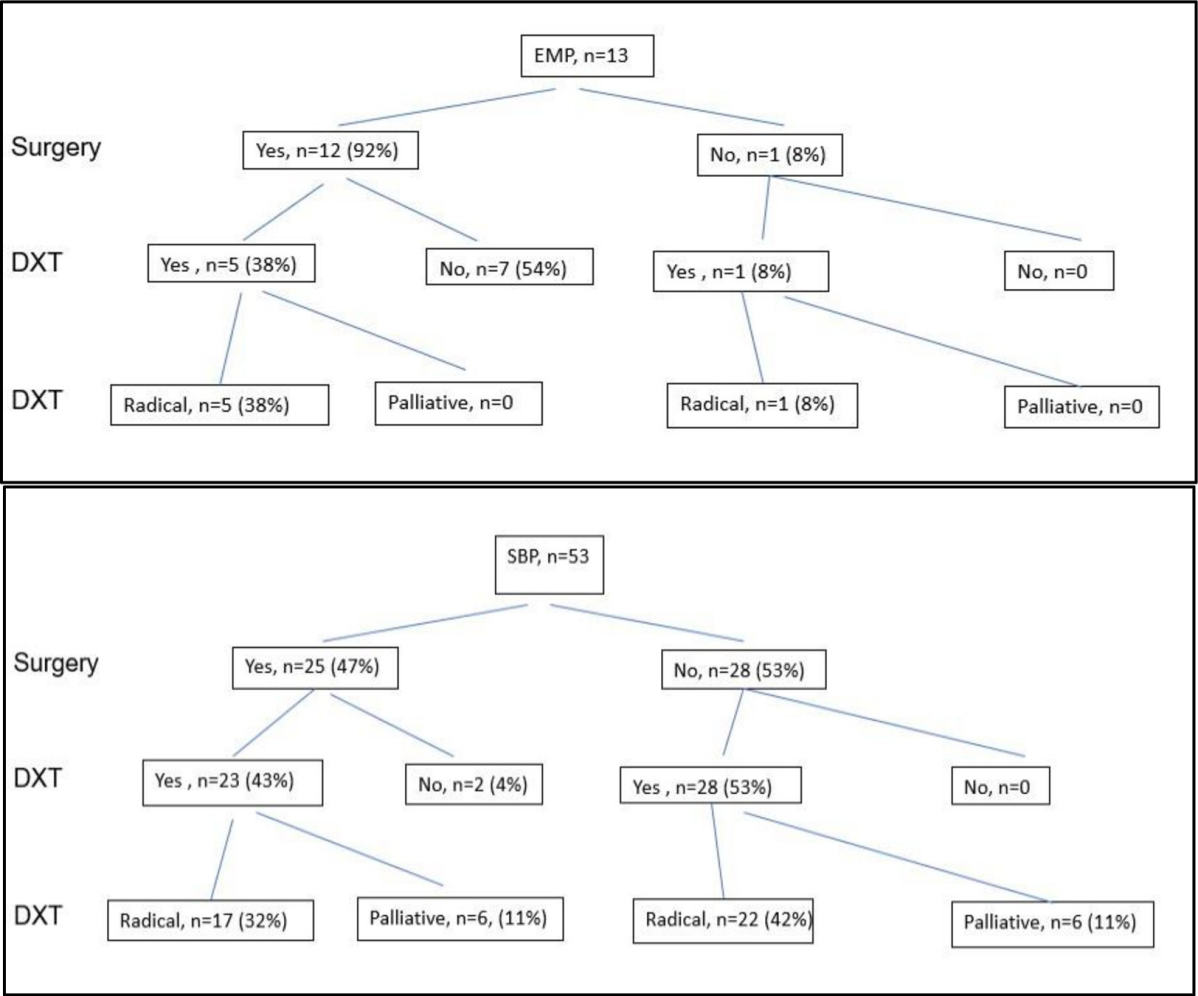
**(A)** SBP treatment pathways (B) EMP treatment pathways.

The treatment pathways of the patients with SBP and EMP are demonstrated in Fig 3. Excision was the sole upfront treatment modality in 7/13 EMP cases and 5/13 EMP cases received RT in addition to surgical resection. No gross imbalance in number of events between these two strategies could be seen but numbers are small (only 4 events). Only 25/53 (47.2%) of bone tumours had surgical treatment as part of the initial management (Fisher’s exact p = 0.004 for comparison of SBPs with EMPs). Radical RT was more commonly used in the treatment of SBP (74%) compared with EMP (46%). Patients with SBP who had surgical treatment tended to receive palliative RT but this difference was not significant (Mann-Whitney p = 0.16). Patients with disease location in the cervical or thoracic spine were more likely to have had surgical treatment than not (Table 2) Surgical treatment of SBPs had no detectable effect on PFS or overall survival.

Only 6 patients received systemic chemotherapy in addition to local treatment (RT +/- surgical) at initial presentation with a strong difference in practice between the 2 centres; Brazil– 5 patients (3 Cyclophosphamide, thalidomide and dexamethasone (CTD), 1 Melphalan-Dexamethasone and 1 combination of Vincristine, Carmustine, Cyclophosphamide, Melphalan and Prednisone (M2); UK– 1 patient attenuated CTD. None of these patients had excess (>5%) plasma cell involvement of their BM at initial staging. All 6 had SBP (1 T-spine, 1 L-spine, 3 pelvis, 1 ribs). UK patient was staged with FDG PET-CT at diagnosis, which revealed a second disease focus, which was an indication for systemic treatment as per the updated IMWG criteria.[3] The 5 Brazilian patients all had initial whole body staging with either CT (4/5 patients) or ^99m^Tc-MIBI (1/5 patients). UK patient progressed to MM 671 days after diagnosis, whereas none of the 5 Brazilian patients relapsed or progressed to MM after a median follow-up of 3755 days (range 424–5110 days).

### Imaging

Whole body/ functional imaging for patients with SP is now a new standard of care as supported by recent guidelines.[15] Baseline whole body imaging with MRI/FDG PET-CT or ^99m^Tc-MIBI was performed in 18/66 cases (27.3%). Functional scans were performed in the FU period after treatment, either based on clinical suspicion or for routine surveillance in 41/66 (62.1%) patients.

In the UK cohort FU imaging was performed in 18/23 patients (78.3%) at a median of 177 days (~ 6 months, IQR 83–237 days) post diagnosis. Reason for follow-up imaging with FDG PET-CT was clinical suspicion of relapse/progression in 6 cases (33.3%), surveillance in 7 cases (38.9%) and unspecified in 5 cases (27.8%). FU imaging revealed no disease progression in 14/18 patients (77.8%), with progressive disease in the remaining 4 patients (22.2%). Three patients had clinical suspicion of progressive disease and reason for scanning is not known for the remaining 1 patient. None of the 7 patients who had FU FDG PET-CT as routine surveillance were upstaged whereas 3 out of 6 patients scanned on grounds of clinical suspicion had positive imaging findings. Of 6 patients scanned because of relapse suspicion (clinical and/or biochemical), 3 with radiological progression on FDG PET-CT received systemic treatment, however all 6 patients ultimately progressed. None of 7 patients known to have had FU FDG PET-CT as routine surveillance had their remission status changed. Five of these patients were monitored only, one was systemically treated and none of these six patients relapsed. The final patient received RT and subsequently progressed.

11 patients were monitored after their first FU FDG PET-CT; none of them had been upstaged by the FU FDG PET-CT, however their outcome appears to differ depending on reason for FU FDG PET-CT scan as 0/5 who had the scan for surveillance progressed in contrast to all three who had the scan because of suspected progression and 2/3 who had the scan for unknown reason. The SUVmax value of the most avid lesion on FU FDG PET-CT in these 11 cases was not found to be useful in predicting outcome (we could not identify any threshold value to discriminate between patients who progressed and those who maintained remission). Diagnostic performance of FU FDG PET-CT was examined against outcome (relapse/progression after FU FDG PET-CT): sensitivity was 40% (12.2–73.8%), specificity 100% (63.1–100%), PPV 100% (39.8–100%), NPV 57.1% (28.9–82.3%), ROC area 0.7 (0.54–0.86).

In the Brazilian cohort 99mTc-MIBI scans were used as an alternative functional imaging technique. FU imaging was performed in 23/43 (53.5%) patients at a median of 467 days (~15 months) (IQR 250–638 days) post diagnosis and revealed no disease progression in 16/23 (69.6%) and progressive disease in 7 patients (30.4%); 13 patients (56.5%) were imaged as part of routine imaging surveillance whilst 10 patients (43.5%) had a clinical suspicion of recurrent or progressive disease. Progressive disease was identified in only 1 patient (7.7%) as part of routine surveillance, and in 6 patients (60%) with a clinical suspicion of recurrent or progressive disease (Fisher’s p = 0.02). Diagnostic performance of FU ^99m^Tc-MIBI scan against actual clinical outcome was similar to FDG PET-CT. Sensitivity was 53.8% (25.1–80.8%), specificity 100% (61.2–100%), PPV 100% (59–100%) and NPV 62.5% (35.4–84.8%), ROC area 0.77 (0.63–0.91).

We analysed diagnostic capacity of first FU functional imaging after combining both cohorts. Positive predictive value was high (100%) whether the scan was done for routine surveillance or for suspected progression. However negative predictive value was low (60%, 95%CI 40.6%-77.3%) and particularly poor (14.3%, 95%CI 0.4–58%) in cases of suspected relapse.

## Discussion

This retrospective analysis of 66 patients with solitary plasmacytoma in the modern era, describes an improved 5 yr OS of 90.7% and a longer median time to progression. We confirm that SBP rather than EMP has a higher risk of progression to MM. Functional imaging at diagnosis prevents misdiagnosis of SP, but the diagnostic utility of routine FU functional imaging appears limited. Treatment modality is not a predictor of survival but adjuvant chemotherapy may be worthy of prospective evaluation.

### Outcomes and risk of progression

The improved survival described in this study (Table 4) was despite a greater proportion of SBP patients when compared with previous studies, suggesting that outcomes are improved in this modern period of analysis. Despite this improved survival, we still observe progression to MM. The risk of progression of SP to MM is considerable, with an incidence of 37–72% and a median time to progression of 2 years^8^. A paucity of patient level data from large cohort studies has resulted in there being no shared consensus about factors are determine risk of progression and no prospective trials have been completed to date in this condition. The pivotal studies to date are outlined in Table 3. One of the largest studies to date was a US population-based study of 2785 patients with SP conducted by El-Fattah et.al.[18] confirming that SBP have a poorer prognosis when compared with EMP, but the study focused only on OS and did not look at risk of progression, imaging nor treatment modality.

**Table 4:** A comparison table of papers from the literature exploring outcomes in solitary plasmacytoma.

	Sharpley et.al (2018)	Finsinger et.al (2015)	Katoditrou et.al (2014)	El-Fattah et. Al (2017)
**Patients (n)**	66	53	97	2785
**Median FU (months)**	53.6	106.8	60	34
**SP location (%) SBP**	83	66	77	74.6
**EMP**	9.7	34	33	25.4
**Risk factors predicting progression to MM/poor survival**	SBP	SBP	Age >60, immunoparesis	Age >60, black-American, SBP
**Median time to progression MM (months)**	61	30	27.5	-
**RT**	86.4	49	43.3	-
**RT+chemo**	9	28	32	-
**Surgery**	56.1	8	7.2	-
**Chemo alone**		15	7.2	-
PFS 5yr (%)	-	73.4	70	-
OS 5 yr (%)	90.7	78.4	84	57

SP = solitary plasmayctoma, MM = multiple myeloma, FU = follow-up, EMP = extramedullary plasmacytoma, PFS = progression free survival, OS = overall survival.

Finsinger et al (2016) analysed the outcomes of 53 Italian patients and confirmed a bone location was a poor prognostic factor, not only of OS, but also of poor PFS, suggesting that a bony location is a RF for progression to MM.[19] Katoditrou et al (2014) found no survival differences between SBP and EMP for either OS or PFS, but that age >60 and a short plasmacytoma free survival rate were predictive of poor OS and immunoparesis for progression to MM.[20] Both papers explore treatment modality in SP but neither report imaging at diagnosis or FU. We confirm that SBP rather than EMP enhances risk of progression to MM, but our data do not confirm previous findings that plasmacytoma size is a risk factor for progression.[10]

### Treatment

We investigated whether the SP treatment strategy influenced clinical outcomes. RT is the preferred treatment modality for plasmacytoma, and our data reflects this with 86.4% of patients being treated with RT, which is higher than that reported by previous groups (see Table 3). RT was used more frequently to treat SBPs (SBP-96.2%, 46.2% EMP) and with a greater proportion (76.5% BP, 46.2% EMP) receiving radical doses (>40GY), although the dose was reduced if surgical resection was performed.

Although many state that surgery is rarely required in the treatment of SP,^2^ our real world data demonstrate the use of surgical resection in 56.1% of cases, the majority of EMP (92.3%, SBP 47.2%). Location influenced the decision to proceed with surgical resection with surgical resection being more frequently for cervical and thoracic location SBP and likely to be for relief of cord compression or stabilisation of the spine. A combined modality approach with surgical resection and RT was used in 42% of cases. With this approach, SBP tended to receive less intense RT doses (p = 0.16). A higher than expected number of PFS events was observed amongst those receiving less than 40Gy, but this may reflect the confounding effect of other clinical variables, but does support work by Mendenhall et. al who previously suggested that a minimum dose of 40Gy is required for optimal control.[21] Our data suggests clinicians should utilise ‘radical’ RT doses of >40 Gy when treating SBP, even after surgical resection, unless limited by patient frailty/co-morbidity until further analysis on the appropriate RT dose for local control of SP is performed.

Systemic chemotherapy is usually deferred until evidence of disease progression. Only 6/66 (9%) in our study received chemotherapy, with 5/6 (83%) remain in first remission with a median follow-up of 3755 days. The role of chemotherapy in the treatment is controversial. Given that treatment modality failed to affect survival, Katoditrou et.al consider chemotherapy to be too toxic and of limited benefit.[20] Finsinger et. Al suggest an approach of chemotherapy after surgery/RT for those patients with SBP but not for EMP given their increased risk of progression to MM.[19] This approach is being evaluated in the prospective IDRIS study, a phase 3 randomised trial of immunomodulatory therapy in high risk SBP.[22]

### Follow-up imaging

Given that treatment modality was not a predictor of survival, we investigated whether improved outcomes could be due to use of functional imaging surveillance. Published data supports the long-felt opinion that whole body imaging is required for the accurate initial diagnosis/staging of patients with SP[22] and IMWG consensus now mandates FDG PET-CT for a suspected diagnosis of SP, provided that WB-MRI is unable to be performed.[3] Although evidence points towards a positive FDG PET-CT scan determining risk of progression to MM, few have explored how and when to image patients and whether we can reliably use FU imaging in this patient group. [23, 24] Our observations do not favour routine surveillance with functional imaging after initial treatment, as the diagnostic yield was low and there is always the risk of nonspecific uptake, however functional imaging can contribute to timely recognition of relapse/progression/evolution to MM in the context of other suspicious clinical symptoms/findings or serology or other laboratory results. Whether this improves outcomes needs to be addressed in a controlled and prospective manner as there is serious risk of bias in retrospective studies like ours.

We also did not find that the maximum standardised uptake (SUVmax) of the most avid lesion correlated with progression risk in this analysis, although previous studies suggest that an SUV lean body mass (SUVlbm) of 5.2 and body surface area (SUVbsa) of >1.7 may correlate with higher risk of transformation.^24^ These alternative biomarkers (maximum standardized uptake value body weight (SUVbw), lean body mass (SUVlbm) may be of value in assessing risk of progression in future.

### Limitations and future direction

This study is not without limitations. Data was collected retrospectively. The study also combines patients from both the UK and Brazil which adds heterogeneity to the study population. Despite variations in diagnostic, therapeutic and follow-up practices and different racial background of patients, outcomes were similar in two large referral centres on the two sides of the Atlantic, which reflects the natural history of the disease and allows validation of findings across populations (Table 4), unlike population based studies, such as the US based study by El-Fattah et. al,[18] and that from the Netherlands,[25] Despite the merging of two cohorts, events were small, but SP are a rare plasma cell dyscrasia and our report reflects real world practice.

The findings described in this study require confirmation within a future case series, or in the planned plasmacytoma trials. Further investigational areas include: correlation between novel imaging and disease features/biology and role for adjuvant chemotherapy in the treatment of SP to improve cure rates. Functional imaging such as FDG PET-CT may have advantages over MRI, if standardised reporting parameters can be agreed, however access to this imaging tool for baseline/follow-up imaging after local treatment did not translate into a meaningful improvement in outcome. The optimal timing of repeat imaging after diagnosis also requires further investigation along with exploring the role of SUV derived parameters, e.g. SUVmax, total lesion glycolysis (TLG) or metabolic tumour volume (MTV) that may enable more accurate prognostication and monitoring of disease response. This study highlights how traditional baseline risk factors for progression are still too imprecise and are difficult to base treatment decisions for individual cases, therefore new biomarkers are needed.

## Conclusion

SPs are a distinct sub-category of plasma cell dyscrasias characterised by their overall good prognosis, but with a varied risk of progression to MM. This study of 66 SP patients from Brazil and the UK confirms a good 5 year OS of 97.5%, with a prolonged PFS of 61 months. SBP rather than EMP enhances risk of progression to MM. Whole body imaging with PET/CT scan at diagnosis may have contributed to more accurate diagnosis of SP, excluding multi-focal/myeloma cases, resulting in selection of patients with better outcomes. Diagnostic utility of routine FU functional imaging appears limited. This study suggests that in cases with suspected disease progression, functional imaging can aid diagnosis, however negative findings cannot reliably exclude impending progression. We hope that this study will encourage a review of current SP guidelines. Treatment modality is not a predictor of survival but adjuvant chemotherapy may be worthy of prospective evaluation.
