# Correction: Long-term clinical outcomes in a cohort of patients with solitary plasmacytoma treated in the modern era

**DOI:** 10.1371/journal.pone.0225184

**Published:** 2019-11-07

**Authors:** F. A. Sharpley, P Neffa, F. Panitsas, T. A. Eyre, J. Kothari, M. Subesinghe, D. Cutter, R. Shcolnik Szor, G. Aparedcida Martinez, V. Rocha, K. Ramasamy

Dr. Toby Eyre should be included in the author byline. He should be listed as the fourth author, and his affiliation is 1: Oxford University Hospital NHS Foundation Trust, Oxford, UK. The contributions of this author are as follows: Curated the data.

The correct citation is: Sharpley FA, Neffa P, Panitsas F, Eyre TA, Kothari J, Subesinghe M, et al. (2019) Long-term clinical outcomes in a cohort of patients with solitary plasmacytoma treated in the modern era. PLoS ONE 14(7): e0219857. https://doi.org/10.1371/journal.pone.0219857
